# Direct oral anticoagulants (DOACs) for postoperative venous thromboembolism prophylaxis in patients with gynecologic malignancies: A quality mini-review

**DOI:** 10.1016/j.gore.2024.101508

**Published:** 2024-09-19

**Authors:** Peter W. Ketch, Sean C. Dowdy, Robert D. McBane, J. Michael Straughn, Teresa K.L. Boitano

**Affiliations:** a Division of Gynecologic Oncology, University of Alabama at Birmingham, Birmingham, AL, USA; b Division of Gynecologic Oncology, Mayo Clinic, Rochester, MN, USA; c Department of Cardiovascular Medicine, Mayo Clinic, Rochester, MN, USA

**Keywords:** Venous thromboembolism (VTE) prophylaxis, Postoperative care, Direct oral anticoagulants (DOACs)

## Abstract

Venous thromboembolism (VTE) is a common cause of morbidity and mortality in gynecologic oncology patients with an increased risk in the postoperative period. Historically, international guidelines have recommended 28 days of low molecular weight heparin (LMWH) or unfractionated heparin (UFH) for extended VTE prophylaxis after major abdominal and pelvic surgery for gynecologic malignancies. Direct oral anticoagulants (DOACs) have emerged as an attractive alternative to injectable anticoagulants. This quality mini-review evaluated the literature around the use of DOACs for postoperative VTE prophylaxis after surgery for gynecologic cancer. Overall, the reviewed literature supports the use of DOACs in select patients within this population which may lead to an improved patient experience, higher rates of treatment compliance, and increased cost savings.

## Highlights

• VTE is a common cause of morbidity and mortality in gynecologic oncology patients.
• DOACs have been established as safe compared to LMWH and UFH for postoperative VTE prophylaxis.
• DOAC use for postoperative anticoagulation leads to improved compliance, patient experience, and cost-effectiveness.
• Gynecologic Oncologists should consider DOACs for extended postoperative VTE prophylaxis in patients after laparotomy.

Venous thromboembolism (VTE) occurs in up to 25–38% of patients with gynecologic cancer and is a leading cause of morbidity and mortality in this population. (Kushnir and Diaz-Montes, 2013, Cohen et al., 2017) In addition to the increased risk of thrombosis due to hypercoagulability of malignancy, gynecologic cancer patients often have additional risk factors including advanced age, reduced mobility, prolonged vascular access, chemotherapy, and obesity. (Gressel et al., 2021, Heit et al., 2000) Cancer-associated thrombosis is associated with decreased quality of life, significant delays in cancer therapy, and decreased overall survival. (Wagner et al., 2019, Abu Saadeh et al., 2013) Furthermore, treatment of VTE is estimated to cost the healthcare system $7 to $10 billion dollars annually. (Grosse et al., 2016) Optimization of postoperative anticoagulation strategies in gynecologic oncology is of great importance recognizing divergent standards of care across healthcare systems. In this quality review, we will discuss the use of direct oral anticoagulants (DOACs) as postoperative VTE prophylaxis in patients with gynecologic malignancies.

Following surgery for malignancy, the risk of VTE is twice that of patients undergoing benign surgeries.(Gressel et al., 2021, Heit et al., 2000, Boo et al., 2022) In the immediate postoperative setting, the risk of VTE varies by gynecologic cancer type with ovarian cancer patients having the highest risk (3.0 %) compared to uterine cancer (1.4 %), cervical cancer (1.5 %), and vulvar cancer (1.2 %).(Graul et al., 2017) Previous studies have demonstrated the use of extended duration (28 days), postoperative anticoagulation with low molecular weight heparin (LMWH) or unfractionated heparin (UFH), decreases VTE rates in the high-risk gynecologic cancer population.(Schmeler et al., 2013, Li et al., 2021, Bergqvist et al., 2002) International guidelines have traditionally recommended the use of LMWH or UFH for extended duration anticoagulation after laparotomy for gynecologic cancer; however, there is increasing interest in the use of DOACs as postoperative VTE prophylaxis.(Gressel et al., 2021, Key et al., 2023).

DOACs act by directly and irreversibly inhibiting factor Xa or factor IIa (thrombin). This class of drugs includes direct factor Xa inhibitors (apixaban, rivaroxaban, edoxaban) and a direct thrombin inhibitor (dabigatran). DOACs have several drug characteristics which make them an attractive anticoagulant compared to LMWH and UFH including: oral administration, availability as a fixed dose regimen, relatively rapid onset of action, no required laboratory monitoring, and no risk of heparin-induced thrombocytopenia. LMWH and UFH are parenterally administered, requiring either daily or twice daily injections, and sometimes necessitate laboratory monitoring. Significant concerns exist regarding patient compliance with these medications in the postoperative setting with 86 % of patients in one study reporting a preference for an oral medication.(Marchocki et al., 2019) Another study reported higher patient satisfaction and decreased perception of the burden of treatment with DOACs compared to LMWH.(Schrag et al., 2021) Furthermore, the COSIMO (Cancer associated thrombosis-patient reported outcomes with rivaroxaban) study reported higher patient satisfaction and compliance with rivaroxaban compared with LMWH.(Cohen et al., 2021) Finally, a cost-effectiveness analysis comparing apixaban with LMWH demonstrated that apixaban is more cost-effective for the prevention of VTE in gynecologic cancer patients.(Glickman et al., 2020).

Given these advantages of DOACsover the previously established standards for postoperative anticoagulation, LMWH and UFH, large trials have been conducted to assess the safety and efficacy of DOACs for VTE prophylaxis after gynecologic oncology surgery. Two recent randomized trials have demonstrated efficacy and safety of DOACs for extended duration postoperative anticoagulation for gynecologic oncology patients. The first was a multicenter, randomized trial by Guntupalli et al, that enrolled 400 patients undergoing surgery for suspected or confirmed gynecologic cancer to either postoperative oral apixaban (2.5 mg twice daily) or subcutaneous enoxaparin (40 mg daily). There was no difference detected in the study’s primary outcome, major bleeding events, (0.5 % vs 0.5 %, p= >0.99). Furthermore, there was no statistically significant difference reported between oral apixaban and subcutaneous enoxaparin in important secondary objectives including clinically relevant nonmajor bleeding events (5.4 % vs 9.7 %), radiologically confirmed VTE (1.0 % vs 1.5 %), medication adherence, or quality of life.(Guntupalli et al., 2020) Of note, this study was underpowered to detect a difference in VTE rates. Patients in the apixaban group reported significantly higher satisfaction compared with the enoxaparin group, specifically regarding the ease of taking the medication, 98.9 % vs 58.8 %, p= <0.001, and less pain associated with taking the medication, 2.1 % vs 49.2 %, p= <0.001.(Guntupalli et al., 2020).

The VALERIA trial, the Venous thromboembolism prophylaxis after gynecoLogical pElvic cancer surgery with Rivaroxaban versus enoxAparin, was a randomized, controlled trial evaluating the efficacy and safety of rivaroxaban versus enoxaparin for VTE prophylaxis after major gynecologic cancer surgery that found no significant difference between VTE rates in patients assigned to rivaroxaban versus enoxaparin, 3.51 % vs 4.39 %, p = 0.7344.(Longo de Oliveira et al., 2022) Of note, the VALERIA trial was stopped early due to lower-than-expected VTE events and did not reach the intended sample size, which limits power. Despite this, the VALERIA trial provides more data to support the use of rivaroxaban for VTE prophylaxis in this high-risk population.(Longo de Oliveira et al., 2022).

While these two randomized controlled trials to date are underpowered to detect differences in postoperative VTE rates between DOACs and enoxaparin, there are no concerning safety signals from these trials to suggest higher rates of VTE in gynecologic oncology patients prescribed DOACs for extended postoperative prophylaxis. Taken together, these trials support DOACs as a safe, easier to administer alternative for postoperative VTE prophylaxis in patients after gynecologic cancer surgery. Larger, adequately powered studies are warranted to validate these findings.

A retrospective study evaluating 315 patients receiving either enoxaparin or rivaroxaban for extended VTE prophylaxis after gynecologic oncology surgery revealed no significant difference in VTE incidence at 30 days (1.7 % in the enoxaparin group vs 1.2 % in the rivaroxaban group, p = 1.0) and reported no significant difference in rates of major bleeding between the two groups (0.4 % in the enoxaparin group vs 3.7 % in the rivaroxaban group, p = 0.06).(Swaroop et al., 2021) Another retrospective cohort study found no difference between VTE rates and postoperative readmissions between patients discharged with extended duration enoxaparin versus apixaban thromboprophylaxis after undergoing laparotomy for gynecologic malignancy, 4 % vs 3 %, p = 0.49 and 5 % vs 6 %, p = 0.50, respectively.(Spenard et al., 2023) A recent systematic review evaluating the use of DOACs for the management of VTE and atrial fibrillation in patients with urogenital and/or gynecologic malignancy explored 7 studies (2 randomized controlled trials and 5 prospective cohort studies with 2 of these trials enrolling patients with atrial fibrillation).(Al-Tourah et al., 2024) The authors report a pooled major bleeding rate of 2.1 %, which is similar to that of the general cancer population.(Al-Tourah et al., 2024).

These results informed the updated Society for Gynecologic Oncology (SGO) clinical practice statement which supports consideration of apixaban for extended postoperative VTE prophylaxis in patients with gynecologic cancer.(Gressel et al., 2021, Key et al., 2023) Importantly, the Guntupalli study included patients who underwent both laparotomy (79.3 %) or laparoscopy (20.8 %) but did not address the question of whether or not gynecologic oncology patients undergoing minimally invasive surgery (MIS) should receive extended duration prophylaxis.(Guntupalli et al., 2020) Due to this, the SGO clinical practice statement recommends individualization of this decision based on patient specific risk factors.(Gressel et al., 2021) Enhanced recovery after surgery (ERAS®) guidelines suggest that extended prophylaxis offers little benefit for patients undergoing MIS, recognizing VTE rates are < 1 %.(Swift et al., 2022, Nelson et al., 2023) While the overall safety of DOACs has been established, consideration must be given to patients with increased bleeding risk or clinical scenarios that may impact the absorption and/or metabolism of these drugs (Table 1).

**Table 1:** Clinical considerations for the use of DOACs that may be encountered in gynecologic oncology patients. (Gressel et al., 2021, Key et al., 2023, Nelson et al., 2023, Talerico et al., 2024, Rosovsky et al., 2023).

	Clinical Consideration**s**
**Increased Bleeding Risk**	
High-risk tumor types	DOACs are not recommended in patients with unresected, high bleeding risk cancers such as many gastrointestinal tumors, intracranial tumors, or renal cancer.
Gastrointestinal disease	DOACS are not recommended in patients with gastric or duodenal ulcers, angiodysplasia, gastrointestinal lesion, previous variceal bleed, treatment related mucosal toxicity, or gastritis, colitis, or esophagitis.
Concurrent use of drugs affecting bleeding risk	Concurrent use of antiplatelet agents should be evaluated and discontinued unless strongly indicated.
Thrombocytopenia	DOACs are not recommended in patients with thrombocytopenia (<50,000 platelets/mL).
Hepatic impairment	Functional hepatic impairment can lead to coagulopathy and associated bleeding events. None of the DOACs are recommended in Child-Pugh Class C. Apixaban should be used with caution in Child-Pugh Class A and B. Rivaroxaban is contraindicated in Child-Pugh Class B and C.
**Altered Metabolism and Absorption**	
Altered gastrointestinal anatomy	Consider parenteral agent in patients with history of significant gastrointestinal surgery (ie: gastric resection or Roux en Y anatomy) or absorption deficiencies. Rivaroxaban and edoxaban impacted most by gastric resection, apixaban mostly absorbed in the small bowel. Consider monitoring plasma DOAC levels.
Nausea and vomiting/inability to tolerate PO	Recommend parenteral agents in patients unable to tolerate PO intake.
Drug-drug interactions	Recommend checking drug-drug interactions prior to prescribing a DOAC. Rivaroxaban and apixaban should not be used with potent inhibitors or inducers of P-glycoprotein (ketoconazole, itraconazole, erythromycin, azithromycin, and clarithromycin) or cytochrome (CYP) P450 3A4 inhibitors (protease inhibitors) or strong CYP 3A4 inducers (rifampicin, carbamazepine, or phenytoin).
Renal impairment	No manufacturer recommended dosage adjustment are recommended for apixaban, however patients with serum creatinine > 2.5 mg/DL or CrCl < 25 mL/min were excluded from clinical trials. The other DOACs have drug specific dose modifications for renal impairment.
Obesity (>120 kg and/or > 40 kg/m2)	Recommend against the use of dabigatran and edoxaban. Rivaroxaban and apixaban may be used with caution, given concerns for reduced drug absorption. Plasma DOAC levels are not recommended to be followed routinely in obese patients, though some experts choose to do so. Current evidence suggests no proven correlation between DOAC drug levels and clinical efficacy and safety.
Low Body Weight (<60 kg)	Recommend avoiding dabigatran and rivaroxaban. Recommend dose adjustments for apixaban and edoxaban, given increased systemic exposure.

In conclusion, pharmacologic VTE prophylaxis is indicated after laparotomy for gynecologic malignancy given high rates of thromboembolic events in this high-risk population. Historically, guidelines have established LMWH and UFH as preferred agents for postoperative thrombophylaxis. Oral DOACs have emerged as a favorable alternative to these injectable medications, especially with the potential for improved treatment compliance, cost-effectiveness, improved patient experience, and established safety. Please see Table 2 for a summary of relevant studies evaluating DOACs vs enoxaparin for VTE thromboprophylaxis in patients undergoing gynecologic cancer surgery. The use of DOACs, specifically apixaban, for extended, postoperative VTE prophylaxis appears to be safe, and effective for selected gynecologic oncology patients.

**Table 2:** Summary of relevant studies evaluating DOAC vs enoxaparin for VTE thromboprophylaxis in patients undergoing gynecologic cancer surgery.

	Study Design	Patient Population	Agents	Primary Outcome(s)	Secondary Outcomes
Guntupalli et al. (Guntupalli et al., 2020)	Randomized Controlled Trial	Patients who underwent major gynecological cancer surgery (79.3 % open surgery)	28 days of apixaban (2.5 mg BID, oral) vs 28 days of enoxaparin (40 mgdaily,subcutaneous)	**Major bleeding events**: 1 patient (0.5 %) vs 1 patient (0.5 %); OR 1.04; 95 % CI 0.07–16.76; p > 0.99	**VTE events**: 2 patients (1.0 %) vs 3 patients (1.5 %); OR 1.57; 95 % CI 0.26–9.50; p = 0.68
				**Clinically relevant nonmajor bleeding events**: 12 patients (5.4 %) vs 19 patients (9.7 %); OR 1.88; 95 % CI 0.87–4.1; p = 0.11	**Adverse events, medication adherence, quality of life**: no significant difference
					**Participant satisfaction, ease of taking medication:** 186 patients (98.9 %) vs 110 patients (58.8 %); OR, 0.06; 95 % CI, 0.01–0.25; p < 0.001. **Participant satisfaction, pain associated with taking the medication:** 4 patients (2.1 %) vs 92 patients (49.2 %); OR 9.20; 95 % CI 2.67–31.82; p < 0.001
The VALERIA Trial (Longo de Oliveira et al., 2022)	Randomized Controlled Trial	Patients who underwent major gynecological cancer surgery (99 % open surgery)	30 days of rivaroxaban (Schmeler et al., 2013) vs 30 days of enoxaparin (40 mg, daily, subcutaneous	**Composite of symptomatic, objectively confirmed VTE and asymptomatic ultrasonography confirmed DVT or VTE related death at 30 days postoperative**: 4 patients (3.51 %) vs 5 patients (4.39 %); RR 0.80, 95 % CI 0.22–2.90; p = 0.7344	
				**Combination of major bleeding plus clinically relevant nonmajor bleeding at 30 days postoperative:** 0 patients (0 %) vs 3 patients (2.63 %); HR 0.14; 95 % CI 0.007–2.73; p = 0.1963	
Swaroop et al. (Swaroop et al., 2021)	Retrospective Cohort	Patients who underwent laparotomy for gynecologic malignancy	Enoxaparin vs Rivaroxaban	**Incidence of VTE, 30 days postoperatively:** 4/233 patients (1.7 %) vs 1/82 patients (1.2 %), p = 1.00.	**Incidence of major bleeding events**: 1/233 patients (0.4 %) vs 3/82 patients (3.7 %), p = 0.06
				**Incidence of VTE, 90 days postoperatively:** 10/233 patients (4.3 %) vs 2/82 patients (2.4 %), p = 0.74	
Spénard et al. (Spenard et al., 2023)	Retrospective Cohort	Patients who underwent laparotomy for gynecologic malignancy	28 days of enoxaparin (40 mgdaily,subcutaneous) vs 28 days of apixaban (2.5 mg BID, oral)	**Total VTE (PE or DVT):** 6 patients (4 %) vs 3 patients (3 %), p = 0.49	**Postoperative Readmissions:** 7 patients (5 %) vs 7 patients (6 %), p = 0.50
				**Incidence of DVT:** 1 patient (1 %) vs 3 patients (3 %), p = 0.27	
				**Incidence of symptomatic PE:** 5 patients (3 %) vs 0 patients (0 %), p = 0.12	

## Declaration of competing interest

The authors declare that they have no known competing financial interests or personal relationships that could have appeared to influence the work reported in this paper.
